# Adaptive Sliding Mode with Finite-Time Convergence for Synchronized Hydraulic Multi-Arm Systems

**DOI:** 10.3390/s26051567

**Published:** 2026-03-02

**Authors:** Bo Gao, Fuqiang Yang, Guangwei Ji, Guanghai Yang, Yuliang Lin, Liangsong Huang

**Affiliations:** 1 Robot Research Center, Shandong University of Science and Technology, Qingdao 266590, China; gaobo@sdust.edu.cn (B.G.); 15621050520@163.com (F.Y.); 15963966307@163.com (G.J.); linyuliangasd@126.com (Y.L.); 2 School of Electrical and Electronic Engineering, Changchun University of Technology, Changchun 130022, China; yangguang1918@163.com

**Keywords:** FTSMC, hydraulic multi-arm systems, synchronized deployment, disturbance rejection, leader–follower framework, confined environments

## Abstract

This study introduces a novel robust finite-time adaptive sliding mode control (FTSMC) strategy, emphasizing its contributions to the synchronized deployment of hydraulically actuated multi-arm systems in confined environments, such as coal bunker cleaning. Key innovations include the integration of adaptive sliding mode control with guaranteed finite-time convergence, a distributed leader–follower framework, and a graph-theoretical communication topology for localized interactions. Specifically, we developed a dynamic model for a multi-agent system comprising one leader and multiple followers, incorporating nonlinear dynamics and unknown external disturbances. The proposed controller ensures rapid finite-time convergence of tracking errors while maintaining robustness against parameter uncertainties, frictional forces, and external perturbations. The theoretical analysis, based on Lyapunov stability, rigorously proves the boundedness and convergence of all system states. Simulation results on a three-arm robotic platform validate the method’s superiority, demonstrating higher tracking accuracy, faster convergence, and stronger disturbance rejection compared with baseline controllers, including SMC, ETASMC, PID, Fixed-Time Consensus Control (FTCC), Disturbance Observer-Based Control (DOBC), and Adaptive Sliding Mode Control (ASMC). This research provides a practical and scalable solution for multi-arm coordination in unstructured environments, significantly advancing the autonomy and reliability of industrial robotic systems.

## 1. Author Summary

In confined and hazardous environments, such as coal bunkers, cleaning operations are often dangerous and labor-intensive. To improve safety and efficiency, robots equipped with multiple hydraulic arms can be used to support and stabilize cleaning platforms. However, coordinating the movement of these arms is challenging due to their nonlinear behavior and unpredictable disturbances, such as friction and load changes.

In this study, we developed a new control method that enables multiple hydraulic arms to work together in a precise and synchronized way. The approach uses a leader–follower strategy, where one arm sets the target motion and the others follow, adapting in real time to disturbances. The method ensures finite-time convergence and strong robustness while maintaining a simple implementable structure suitable for engineering deployment.

Our method ensures that all arms reach the desired position quickly and remain stable, even under strong interference. Simulations using a three-arm robot model show that our control strategy outperforms traditional methods in speed, accuracy, and robustness. This work contributes to safer and more reliable robot deployment in real-world industrial settings.

The simulations in this study were implemented using MATLAB R2021a (The MathWorks, Inc., Natick, MA, USA).

## 2. Introduction

Hydraulically actuated robotic arms are widely used in industrial tasks requiring high power density, accurate force control, and robustness in harsh environments. Meanwhile, multi-agent consensus and coordination theory has become a fundamental framework for cooperative tracking and synchronization in networked robotic systems [1]. In industrial automation, hydraulic manipulator control has therefore received increasing attention, particularly for reliable operation under complex dynamics and uncertain environments [2,3]. For multi-arm hydraulic platforms, fault-tolerant and cooperative control strategies have been explored to improve safety and reliability, and optimal control design has also been investigated to enhance deployment performance [4,5].

A typical application involves multiple hydraulic arms working in unison to stabilize a central platform, where synchronous deployment is critical. Leader–follower formation control and prescribed-performance coordination are common approaches to guarantee tracking behavior under specified transient and steady-state constraints [6,7,8]. To further improve convergence speed and robustness, robust finite-time and terminal sliding mode control methods have been developed for nonlinear systems with disturbances [9,10,11,12], including non-singular terminal sliding mode designs and finite/fixed-time stability results [13,14]. In addition to sliding-mode-based solutions, distributed model predictive control has been used to address cooperative coordination via local optimization and neighbor interaction, though it may be sensitive to model mismatch and computational cost in strongly nonlinear hydraulic settings [15]. Recently, learning-based distributed leader–follower control (e.g., recurrent SAC structures) has also been investigated to enhance adaptivity under uncertainty, while stability guarantees and interpretability remain challenging for complex dynamics [16]. From a theoretical perspective, finite-time consensus for networked Euler–Lagrange systems and prescribed-time/finite-time leader–follower consensus provide supporting tools for time-guaranteed synchronization under limited local information and directed topologies [17,18,19]. Related distributed finite-time coordination in attitude/rigid-body systems further enriches distributed stability analysis for multi-robot synchronization [20,21], and distributed formation/consensus control has been studied for general multi-agent systems to improve scalability [22,23].

However, practical multi-arm hydraulic coordination still faces notable gaps. First, strong nonlinearities caused by fluid dynamics, friction, load variability, and pressure fluctuations, together with disturbances, delays, and constrained communication, can significantly degrade synchronization performance. Second, safety requirements such as collision avoidance and non-communicating constraints may complicate cooperative deployment in confined spaces [24]. Third, noise, time delays, and even attacks have been shown to deteriorate formation/consensus performance, motivating stronger robustness mechanisms for adverse and uncertain environments [25,26,27]. Although distributed nonlinear trajectory optimization can scale to larger systems [28], maintaining finite-time guarantees under intensified uncertainties remains difficult for sliding-mode-based consensus controllers in discrete-time/continuous-time settings [29]. Event-triggered terminal sliding mode methods can improve efficiency in networked systems, but robust finite-time coordination under realistic hydraulic uncertainties still needs further study [30]. Moreover, cooperative robotics studies highlight the importance of localized interaction, reliable information exchange, and coordinated control under actuator dynamics [31,32]. Classical variable-structure theory continues to underpin sliding-mode baselines [33], and observer-based disturbance compensation (e.g., DOBC and integral sliding-mode observers) provides systematic enhancement of disturbance rejection [34]. In industrial practice, PID remains a widely accepted benchmark for coordinated motion control [35], and fault-tolerant cooperative control surveys further emphasize robustness requirements in safety-critical environments [36].

Motivated by these gaps, this paper develops a robust finite-time adaptive sliding-mode coordination framework for hydraulically actuated multi-arm systems under a leader–follower architecture. The proposed method aims to guarantee finite-time (or practical finite-time) convergence of coordination errors, ensure boundedness of closed-loop signals under parameter variations and external disturbances, and achieve high-precision synchronization in both position and velocity using only localized neighbor information. Extensive simulations on a tri-arm system are conducted under disturbance injection and parameter perturbations mimicking hydraulic friction/load variability, and performance is evaluated using unified indices (tracking RMSE, recovery/settling speed, and cumulative control effort) with comparisons to representative baselines.

The main contributions are summarized as follows:

(1) A leader–follower distributed finite-time adaptive sliding-mode coordination controller is developed for multi-arm hydraulic systems to achieve fast position/velocity synchronization.
(2) A localized-interaction distributed architecture is formulated under graph-theoretic communication to improve scalability and reduce information requirements.
(3) Lyapunov-based analysis is provided to establish boundedness and finite-time (or practical finite-time) reaching properties with explicit convergence characterization.
(4) Comprehensive simulations under disturbance/parameter perturbations validate robustness and quantify accuracy–speed–effort trade-offs against classical and representative baselines.

The remainder of this paper is organized as follows. Section 3 presents modeling and controller design with stability analysis. Section 4 extends the method to multi-cylinder height/position synchronization with finite-time convergence analysis. Section 5 and Section 6 report results and comparisons, and Section 7 concludes the paper.

## 3. Angle Coordination Control

### 3.1. System Description

In the initial deployment stage, a robotic platform equipped with three hydraulic arms was required to perform a synchronized unfolding action. The objective was to ensure that all arms gradually expand in coordination, forming a stable triangular configuration with 120° angular spacing and a −10° inclination with respect to the horizontal plane. Each arm consists of two joints: a primary swinging joint and a support foot swinging joint.

In this section, we focus on the coordinated control of these two joint angles to maintain angular consistency among all robotic arms. The multi-joint coordinated control of hydraulic arms is critical for structural stability in hazardous environmental applications [3], and angular consistency directly affects the reliability of the support platform.

### 3.2. Dynamic Modeling

For clarity, the main symbols and parameters used in the Euler–Lagrange dynamic model are summarized in Table 1. 

Consider a multi-agent system consisting of N + 1 hydraulically actuated robotic arms, indexed from 0 to N, where agent 0 is the leader and agents 1 through N are followers [1]. The dynamics of each robotic arm are governed by the following Euler–Lagrange Equation (1):

(1) $$M_{i}(q_{i}){\ddot{q}}_{i}+C_{i}(q,\dot{q}){\dot{q}}_{i}+G_{i}(q_{i})=\tau_{i},i=1\ldots N$$

where:

$M_{i}(q_{i})$: symmetric, positive definite inertia matrix

$C_{i}(q,\dot{q})$: Coriolis and centrifugal forces

$G_{i}(q_{i})$: gravitational term

$\tau_{i}$: control input

We made the following standard assumptions on the system:

**Property** **1.** $M_{i}(q_{i})$ *is a symmetric positive definite matrix and satisfies * $m_{1}I\leq M_{i}(q_{i})\leq m_{2}I$.

**Property** **2.** *The matrix* ${\dot{M}}_{i}(q_{i})-2C_{i}(q_{i},{\dot{q}}_{i})$ *is skew-symmetric.*

**Property** **3.** *Non-negative constants (* $M_{max},C_{max},G_{max}$ *) exist, such that:*

(2) $$∥M_{i}(q_{i})∥\leq M_{max},∥C(q_{i},{\dot{q}}_{i}){\dot{q}}_{i}∥\leq C_{max}∥{\dot{q}}_{i}∥,∥G_{i}(q_{i})∥\leq G_{max}$$

To facilitate controller design, we converted the dynamics into a first-order state-space form:

Define the following:

(3) $$x_{i}=q_{i},v_{i}={\dot{q}}_{i}$$

Then, the state-space equations become the following:

(4) $${\dot{x}}_{i}=v_{i}$$

(5) $${\dot{v}}_{i}=M_{i}^{-1}(q_{i})(\tau_{i}-C_{i}(q_{i},{\dot{q}}_{i}){\dot{q}}_{i}-G_{i}(q_{i}))=u_{i}+f_{i}$$

We defined the following:

(6) $$u_{i}=M_{i}^{-1}(q_{i})\tau_{i}$$

(7) $$f_{i}=M_{i}^{-1}(q_{i})(-C_{i}(q_{i},{\dot{q}}_{i}){\dot{q}}_{i}-G_{i}(q_{i}))$$

### 3.3. Assumptions and Lemma

**Assumption** **1.** *The leader’s trajectory and its derivatives are bounded:*

(8) $$∥q_{0}(t)∥,∥{\dot{q}}_{0}(t)∥,∥{\ddot{q}}_{0}(t)∥\leq {\bar{\varpi }}_{0},\forall t\in [0,\infty )$$

**Assumption** **2.** *The leader’s position is constrained:*

(9) $$k_{p}<b_{0_{p}}(t)\leq q_{0p}(t)\leq {\bar{b}}_{0p}<{\bar{k}}_{p}$$

**Lemma** **1.** *Given Properties 1 and 2,* $f_{i}$ *is bounded, i.e.,* $∥f_{i}∥\leq f_{\max}$.

### 3.4. Error System Design

In order to analyze and design a distributed coordination control strategy for the hydraulically actuated multi-arm system, it was necessary to characterize the deviation of each follower agent from the leader and from its neighbors. To this end, this section introduces the error system formulation, which includes both individual tracking errors and local consensus errors. These errors form the basis for constructing the sliding surfaces and designing the cooperative controller. By defining the tracking and consensus errors in a matrix form, a compact and scalable representation of the overall coordination error is obtained, which facilitates subsequent stability analysis and controller synthesis.

Defined the tracking errors as follows:

(10) $$\begin{array}{l} {\tilde{x}}_{i}=q_{i}-q_{0}=x_{i}-x_{0},\\ {\tilde{v}}_{i}={\dot{q}}_{i}-{\dot{q}}_{0}=v_{i}-v_{0}, \end{array}$$

We defined the local consensus errors as follows:

(11) $$e_{xi}=\sum_{j=1}^{N}a_{ij}(x_{i}-x_{j})+b_{i}(x_{i}-x_{0})$$

(12) $$e_{vi}=\sum_{j=1}^{N}a_{ij}(v_{i}-v_{j})+b_{i}(v_{i}-v_{0}),i=1\ldots N$$

It then follows that:

(13) $$e_{vi}={\dot{e}}_{xi}$$

Let the stacked vectors of local consensus errors be defined as follows:

(14) $$e_{x}={\left[e_{x1}^{T}\ldots e_{xN}^{T}\right]}^{T},\;e_{v}={\left[e_{v1}^{T}\ldots e_{vN}^{T}\right]}^{T}$$

(15) $$\tilde{x}={\left[{\tilde{x}}_{1}^{T},\ldots ,{\tilde{x}}_{N}^{T}\right]}^{T},\tilde{v}={\left[{\tilde{v}}_{1}^{T},\ldots ,{\tilde{v}}_{N}^{T}\right]}^{T}$$

Then, the compact representation of the total tracking error is expressed as follows:

(16) $$\begin{array}{l} e_{x}=(L+B)⊗I_{n}\cdot \tilde{x}\\ e_{v}=(L+B)⊗I_{n}\cdot \tilde{v} \end{array}$$

The dynamics of the aggregated consensus error system can thus be written as follows:

(17) $$\begin{array}{l} {\dot{e}}_{x}=e_{v}\\ {\dot{e}}_{v}=(L+B)⊗I_{n}\cdot \left(u+F-1_{N}⊗f_{0}-1_{N}⊗u_{0}\right) \end{array}$$

where:

(18) $$F={\left[f_{1}^{T}\ldots f_{N}^{T}\right]}^{T},\;u={\left[u_{1}^{T}\ldots u_{N}^{T}\right]}^{T}$$

Since the matrix L + B is invertible, the cooperative consensus tracking problem for the MAS (multi-agent system) is equivalent to the stability of the aggregated tracking error system. This error modeling approach draws on the ideas of adaptive leader–following consensus for Euler–Lagrange systems [21], ensuring consistency in multi-agent coordination. The matrix-form error representation is consistent with distributed formation control via graph theory [22], enhancing the scalability of the control strategy for multi-arm systems.

### 3.5. Controller Design and Stability Analysis

In this section, a consensus control strategy was designed based on adaptive control, sliding mode control, and the Lyapunov theory. The integration of adaptive and sliding mode control has been proven effective for handling nonlinearities and uncertainties in hydraulic systems [1,5], balancing robustness and adaptability.

The design of the sliding mode consensus protocol consists of two main steps:

1. A sliding manifold was constructed such that the multi-agent system (MAS) can achieve consensus when the system states remain on it.
2. A sliding mode control protocol was then developed to drive the system states onto the sliding manifold and maintain motion along it.

### 3.6. Angle Sliding Variable Definition

To avoid confusion, we note that this section defines the sliding variable for the angle coordination stage and serves as the basis for the control protocol derived in Section 3.7. The sliding-surface design in Section 4.6 is developed for the position (height) synchronization stage and adopts a different error construction and design rationale; therefore, it is presented separately and is not repeated here.

We defined the sliding surface for each agent as follows:

(19) $$s_{i}=\mu e_{xi}+e_{vi}$$

where, μ is a positive real number.

In vector form, the total sliding surface can be expressed compactly as follows:

(20) $$s=\mu e_{x}+e_{v}$$

where, $s={\left[s_{1}^{T},\ldots ,s_{N}^{T}\right]}^{T}$.

When the system trajectory is constrained to lie on the manifold s=0, consensus among the agents is achieved. The sliding surface design references the robust integral terminal sliding mode control framework [9], enhancing convergence speed and steady-state accuracy, and aligns with classical sliding surface formulations [33]. Additionally, the vector-form sliding surface is consistent with distributed multi-agent control design [23,29], facilitating coordinated error suppression across all arms.

### 3.7. Control Protocol Design

The derivative of the sliding surface can be written as follows:

(21) $$\dot{s}=\mu {\dot{e}}_{x}+{\dot{e}}_{v}=\mu e_{v}+(L+B)⊗I_{n}\cdot \left(u+F-1_{N}⊗f_{0}-1_{N}⊗u_{0}\right)$$

We propose the following equivalent control law to ensure asymptotic convergence to the sliding manifold:

(22) $$\dot{s}=-K_{1}s-K_{2}sign(s)-\psi_{c}(s)$$

Accordingly, the discontinuous sliding mode consensus protocol is defined as follows:

(23) $$u=\phi \left(-K_{1}s-K_{2}sign(s)+g_{c}(s)-\mu e_{v}\right)+1_{N}⊗u_{0}$$

where the compensation term $g_{c}(s)$ is defined as follows:

(24) $$g_{c}(s)={\left[\psi_{c}{\left(s_{1}\right)}^{T},\ldots ,\psi_{c}{\left(s_{N}\right)}^{T}\right]}^{T}.$$

And each $\psi_{c}(s_{i})$ is a nonlinear gain term expressed as follows:

(25) $$\psi_{c}(s_{i})=-\beta_{i}\frac{s_{i}}{\left\|s_{i}\right\|}$$

The adaptive law for $\beta_{i}$ is as follows:

(26) $${\dot{\beta }}_{i}=\chi (\|s_{i}\|-\gamma \beta_{i}),\;\beta_{i}(0)\geq 0,$$

where ξ>0,χ>0,γ>0. are constant design parameters.

The discontinuous switching action improves robustness but may induce chattering in practical hydraulic implementations. In real systems, the sign function can be replaced by a saturation/tanh function with a small boundary layer to obtain a smooth control input, which leads to finite-time practical reaching while preserving strong disturbance rejection.

If Assumptions 1 and 2 and Lemma 1 hold, then under the action of the adaptive discontinuous sliding mode consensus protocol defined above, the multi-agent system achieves consensus tracking. The adaptive law design was inspired by hybrid gain finite-time sliding mode controllers [11], balancing robustness against disturbances and chattering suppression while incorporating classical adaptive sliding mode techniques [37]. The nonlinear gain term was introduced to enhance disturbance rejection, a common technique in terminal sliding mode control [13,30], with roots in classical sliding mode theory [33].

### 3.8. Stability Analysis

To make the derivation self-contained, we first bound the nonlinear coupling terms using standard Euler–Lagrange properties and Lipschitz-type inequalities, and then we established an asymptotic convergence result for the sliding variable via a Lyapunov comparison argument.

To analyze the stability of the proposed consensus control system, we constructed the following Lyapunov candidate function:

(27) $$V=\frac{1}{2}s^{T}s+\frac{1}{2\chi }\sum_{i=1}^{N}{\left(\beta_{i}-\bar{\beta }\right)}^{2}.$$

Taking the time derivative of V gives the following:

(28) $$\dot{V}=s^{T}\dot{s}+\frac{1}{\chi }\sum_{i=1}^{N}(\beta_{i}-\bar{\beta }){\dot{\beta }}_{i}$$

We substituted the expression of $\dot{s}$ into the equation as follows:

(29) $$\begin{array}{l} \dot{V}=s^{T}\dot{s}+\frac{1}{\chi }\sum_{i=1}^{N}(\beta_{i}-\bar{\beta }){\dot{\beta }}_{i}\\ =s^{T}\left(-K_{1}s-K_{2}sign(s)+g_{c}(s)+(L+B)\left(F-1_{N}⊗f_{0}\right)\right)\\ +\sum_{i=1}^{N}\left(\beta_{i}-\bar{\beta })(\left\|s_{i}\right\|-\gamma \beta_{i}\right) \end{array}$$

To further estimate the nonlinear term $\left\|\left(F-1_{N}⊗f_{0}\right)\right\|$, using Lipschitz continuity of the dynamics and Theorem 1, we derived the following:

(30) $$\begin{array}{l} \left\|\left(F-1_{N}⊗f_{0}\right)\right\|\\ =\left\|{\left[\left\|f(x_{1},v_{1},t)-f(x_{0},v_{0},t)\|\cdots \|f(x_{N},v_{N},t)-f(x_{0},v_{0},t)\right\|\right]}^{T}\right\|\\ \leq \left\|{\left[\zeta_{1}\|{\tilde{x}}_{1}\|+\zeta_{2}\|{\tilde{v}}_{1}\|,\cdots ,\zeta_{1}\|{\tilde{x}}_{N}\|+\zeta_{2}\|{\tilde{v}}_{N}\|\right]}^{T}\right\|\\ \leq \zeta_{1}\|\tilde{x}\|+\zeta_{2}\|\tilde{v}\|. \end{array}$$

Therefore:

(31) $$\|(L+B)⊗I_{n}\cdot (F-f)\|\leq \epsilon (\zeta_{1}\left\|e_{x}\right\|+\zeta_{2}\left\|e_{v}\right\|).$$

Substituting all terms back yields the following:

(32) $$\begin{array}{l} \dot{V}\leq \left[\epsilon (\zeta_{1}\left\|e_{x}\right\|+\zeta_{2}\left\|e_{v}\right\|)\right]\left\|s\right\|\\ +s^{T}\left(-K_{1}s-K_{2}sign(s)+g_{c}(s)\right)+\sum_{i=1}^{N}\left(\beta_{i}-\bar{\beta })(\left\|s_{i}\right\|-\gamma \beta_{i}\right)\\ \leq s^{T}\left(-K_{1}s+g_{c}(s)\right)+\sum_{i=1}^{N}\left(\beta_{i}-\bar{\beta })(\left\|s_{i}\right\|-\gamma \beta_{i}\right) \end{array}$$

Because:

(33) $$s^{T}g_{c}(s)=-\sum_{i=1}^{N}\beta_{i}\left\|s_{i}\right\|$$

we obtained the following:

(34) $$\begin{array}{l} \dot{V}\leq -K_{1}s^{T}s+\sum_{i=1}^{N}\gamma \beta_{i}\bar{\beta }\\ \leq -K_{1}s^{T}s+\gamma \sum_{i=1}^{N}{\bar{\beta }}^{2}\\ \leq -K_{1}s^{T}s+\eta_{1}, \end{array}$$

where, $\eta_{1}=N\gamma {\bar{\beta }}^{2}$.

Based on the above Lyapunov analysis, it follows that all closed-loop signals of the proposed consensus control system remain bounded. Since $\dot{V}\leq -K_{1}s^{T}s+\eta_{1}$, we can conclude that: (1) the sliding variable s and adaptive gain $\beta_{i}$ are uniformly ultimately bounded (UUB) (bounded by $\sqrt{2\eta_{1}/K_{1}}$); (2) by Barbalat’s lemma, $lim_{t\to \infty }∥s(t)∥=0$, which implies the angle tracking error ${\tilde{q}}_{i}$, asymptotically converges to zero (i.e., all hydraulic arms achieve angle synchronization).

Therefore, the proposed distributed controller guarantees consensus tracking performance for the entire multi-agent hydraulic arm system under the considered assumptions. This completes the stability proof and validates the theoretical foundation of the proposed coordination strategy. The stability analysis builds on the Lyapunov stability criteria for sliding mode control [10,12], ensuring rigor of the proposed method, and follows foundational Lyapunov theory [6] for finite-time convergence. Lyapunov-based stability proof is a standard tool in multi-agent consensus control [8,19], providing solid theoretical support for the control strategy, as established in classical texts on nonlinear control [6].

### 3.9. Simulation Results: Angle Tracking

To verify the proposed multi-arm coordination control strategy’s effectiveness and robustness, comprehensive simulations were conducted on a hydraulically actuated robotic platform, emulating realistic complex operational conditions. A multi-step reference input signal was constructed to rigorously evaluate joint angle tracking performance across the full actuation range and examine joint trajectory following over time.

During the simulations, abrupt step changes were introduced at 3, 6, 9, 12, and 15 s to represent sudden command variations; external disturbances were also applied between 9 and 12 s to assess the system’s anti-disturbance capability and stability.

The simulation results and figures show that each joint’s angular positions and velocities maintain high tracking accuracy with minimal reference deviation. Even under external disturbances, the system remains stable with a smooth dynamic response, and the sliding surface converges rapidly to zero—confirming the proposed finite-time sliding mode controller’s fast convergence and strong robustness against disturbances and uncertainties. These results validate the strategy’s practical applicability for real-world robotic coordination [12], and multi-step input tests (commonly used for joint tracking verification [3,4]) ensure its industrial relevance, consistent with classical hydraulic control validation methods [5].

**Figure 1:**
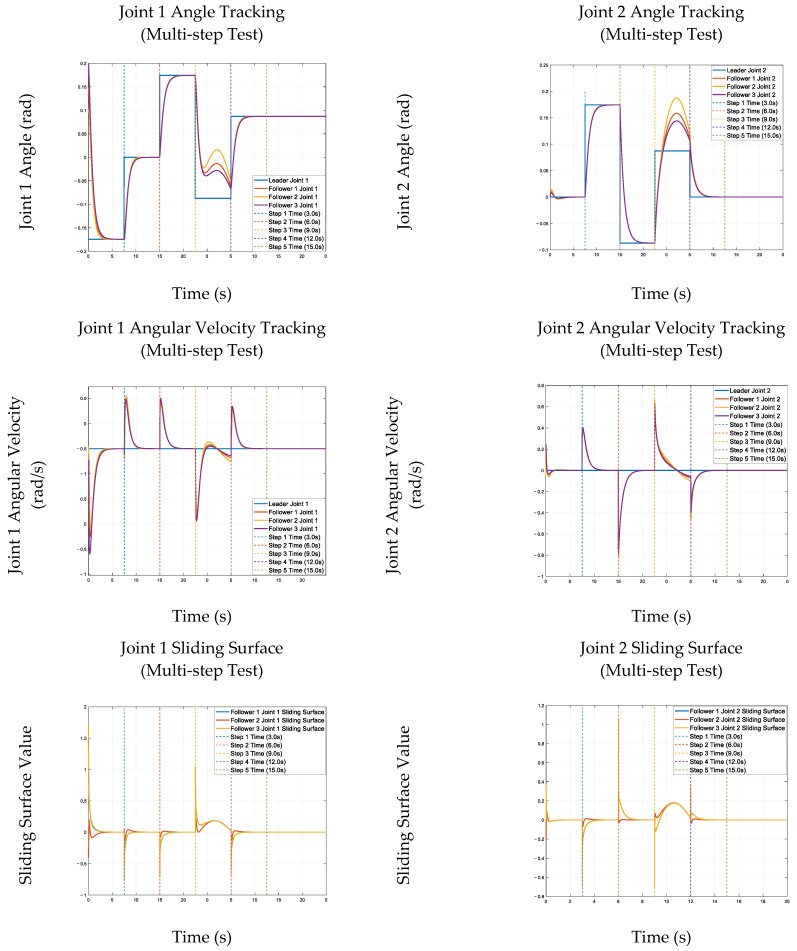
Simulation Results of Joint Angle, Velocity, and Sliding Surface Tracking under Multi-Step Input Conditions. This figure shows the simulation results for joint tracking under sliding mode control, including joint angles, velocities, and sliding surfaces, all demonstrating fast convergence, accurate tracking, and strong robustness to disturbances under multi-step input conditions.

The joint-angle, velocity, and sliding-surface tracking performance under multi-step inputs is illustrated in Figure 1.

## 4. Position Synchronization Control

### 4.1. Modeling Description and Control Objective

This section formulates the position/height synchronization problem for the hydraulically actuated multi-arm system under the leader–follower framework. The objective was to design a distributed controller that enables each follower to track the leader’s reference trajectory while maintaining the prescribed relative offsets, despite modeling uncertainties and external disturbances. For clarity, we first define the tracking/consensus error variables and then construct a finite-time coordination control strategy in the subsequent subsections.

In the fixed support stage, the three already-deployed hydraulic arms are required to reach the same height inside the coal bunker wall at an identical speed. This ensures that the central body of the coal bunker cleaning robot is stably fixed at the center of the workspace. To achieve precise positioning and highly reliable support performance, all support arms must maintain consistency in both position synchronization and velocity synchronization during the movement process, thereby avoiding structural imbalances or misalignments that could affect the stability of the robotic platform. Precise position synchronization of multi-arm hydraulic systems is critical for hazardous environment operations [3], and velocity consistency is essential to prevent structural stress caused by asynchronous movement.

### 4.2. System Modeling and Control Architecture

In this subsection, we present the follower dynamics used for the hydraulic position/height control layer and rewrite the system in a compact form suitable for distributed controller synthesis. To explicitly account for practical effects such as friction, load variation, and unmodeled nonlinearities, the unknown terms are grouped into a lumped uncertainty/disturbance item. This modeling step provides a unified basis for the sliding surface design and finite-time stability analysis that follows.

We begin with a standard nonlinear dynamic model of a single hydraulic actuator and build upon this to model a collaborative multi-cylinder system. Based on a master–slave architecture and multi-agent coordination theory, the system is modeled as a multi-agent network comprising N + 1 hydraulic cylinders. This master–slave framework is consistent with distributed leader–follower formation control based on bioinspired neural dynamics [17], ensuring effective coordination among multiple cylinders. Nonlinear dynamic modeling of hydraulic actuators is a foundational step in multi-arm control design [2,5], capturing key characteristics such as friction, oil compressibility, and load variations. In this configuration, the master cylinder (agent 0) generates a reference trajectory, whereas the N follower cylinders (agents 1 through N) implement distributed control to achieve finite-time tracking, thereby ensuring positional consistency.

Thus, the multi-arm synchronization problem is transformed into the problem of coordinated extension among multiple hydraulic cylinders. Each agent’s motion dynamics can be expressed in second-order form, incorporating nonlinearities such as friction, oil compressibility, and load variations. The system is described as follows:

(35) $${\dot{p}}_{i}=r_{i},{\dot{r}}_{i}=g_{i}(p_{i})+U_{i},i=1,\ldots ,N$$

where $p_{i}$ and $r_{i}$ represent the position and velocity of the i-th follower, $U_{i}$ is the control input, and $g_{i}(p_{i})$ represents the nonlinear disturbances including friction, load dynamics, and internal coupling effects.

The overall control goal was to design a distributed control law such that all follower cylinders achieve finite-time tracking of the leader’s reference trajectory $p_{0}(t)$, while satisfying the following criteria:

(1) Finite-time convergence: the convergence time is bounded and independent of the initial conditions;
(2) Robustness: the system remains stable under external disturbances and abrupt load changes;
(3) Synchronization accuracy: the steady-state error is less than 1 cm, and the peak tracking error remains below 1 cm.

The control criteria are aligned with the performance requirements of hydraulic robotic arms in unstructured environments [4], ensuring practical applicability in industrial scenarios.

### 4.3. Proportional Valve Control Mechanism and Velocity Generation

Since each actuator/arm is only allowed to exchange information with its neighbors, the coordination objective was implemented through a graph-theoretic communication topology. This subsection specifies the neighbor sets and interaction weights and shows how local information is used to construct distributed consensus errors. These definitions ensure that the proposed controller is scalable and can be executed in a fully distributed manner.

To accurately describe the dynamic behavior of the hydraulic actuation system, the velocity control characteristics of the proportional valve were incorporated into the model. Under quasi-steady-state conditions, the piston velocity $r_{i}$ is related to the valve opening $u_{i}$ via the following nonlinear mapping:

(36) ri=Φ(ui,ptarget,i)=(CdAvuiAp)2(ps−ptarget,i)/ρ

Here, $\Phi (u_{i},p_{target})$ denotes the velocity output function of the proportional valve, where $u_{i}$ is the control signal (valve opening ratio) and $p_{target,i}$ is a virtual chamber pressure designed according to the desired motion profile, rather than measured in real-time. Proportional valve control is a key component of hydraulic actuator dynamics [2,3], and its nonlinear characteristics must be considered for precise velocity control in multi-arm coordination.

To formulate the dynamic model of the system, the following state update form was introduced, treating the piston velocity as a controlled dynamic process. Its differential form is given by Equation (1).

(37) $$\begin{array}{l} U_{i}=\dot{\Phi }(u_{i},P_{target,i})\\ =k\sqrt{P_{s}-P_{target,i}}\cdot {\dot{u}}_{i}-\frac{ku_{i}}{2\sqrt{P_{s}-P_{target,i}}}\cdot {\dot{P}}_{target,i} \end{array}$$

Consequently, the complete dynamic equation of the system is given by the following:

(38) $${\dot{r}}_{i}=g_{i}(p_{i})+\dot{\Phi }(u_{i},P_{target,i})$$

Treating velocity as a controlled state is a common technique in finite-time control design [10,30], enhancing the controllability of the system and facilitating fast convergence.

### 4.4. Relevant Definitions

To avoid ambiguity in performance objectives and to make the subsequent analysis self-contained, we introduce the relevant definitions for consensus tracking and finite-time convergence in this subsection. These definitions clarify what is meant by synchronization in position and velocity and provide the formal criteria that will be used to evaluate the proposed control strategy.

The cooperative tracking objective of a hydraulic multi-agent system is to ensure that the position and velocity of all follower cylinders converge to those of the leader cylinder. This ensures that the end-effectors of the robot arms, driven by the hydraulic actuators, reach the same height at the same time. Accordingly, the consensus control problem can be reformulated as the stability analysis of the error dynamics system.

**Definition** **1** **(**[1]**).** *The system is said to achieve asymptotic consensus if the tracking errors of all follower hydraulic cylinders converge to zero, i.e.,* $\underset{t\to \infty }{\lim}\;{\bar{p}}_{i}=0$, $\underset{t\to \infty }{\lim}\;{\bar{r}}_{i}=0$ *, where the position and velocity tracking errors are defined as* ${\bar{p}}_{i}=p_{i}-p_{0}-d_{i},{\bar{r}}_{i}=r_{i}-r_{0}$* . Here,* $d_{i}$ *denotes the desired offset between follower i and the leader, representing the predefined spatial separation to be maintained. Achieving consensus implies that the terminal positions of the robot arms become identical after extension.*

**Definition** **2 (Finite-Time Stability Criterion** [1]**).** *Considering a system* $\dot{x}=f(x)$ *, where the origin is an equilibrium point, if there exists a class-* $C^{1}$ *positive definite Lyapunov function* V(x) *satisfying* $\dot{V}(x)\leq -kV{\left(x\right)}^{\alpha }$*,* k>0*,* 0<α<1*, then the system state will converge to the origin in finite time (T), which is bounded by* $T\leq \frac{V{\left(x(0)\right)}^{1-\alpha }}{k(1-\alpha )}$.

### 4.5. Error System Modeling

With the consensus error system established, we then designed an appropriate sliding surface to encode the coordinated tracking objective. The sliding variable was constructed using local position and velocity errors so that driving it to zero enforces synchronization among all agents. This design also enables a Lyapunov-based analysis to explicitly characterize finite-time reaching behavior.

To uniformly describe the position and velocity errors of the multi-cylinder hydraulic system and to provide a mathematical foundation for subsequent controller design, this paper introduces the adjacency matrix and Laplacian matrix from algebraic graph theory [22], based on the mathematical model defined in Section 2. The coupling relationships between agents are expressed in matrix form, referencing the distributed formation control framework [18,28]. Graph-theoretical tools are essential for modeling distributed multi-agent interactions [17,22], ensuring scalable coordination among multiple hydraulic cylinders. Specifically, the local position and velocity errors are defined as follows:

Local position error:

(39) $$e_{pi}=\sum_{j=1}^{N}a_{ij}({\bar{p}}_{i}-{\bar{p}}_{j})+b_{i}{\bar{p}}_{i}$$

Local velocity error:

(40) $$e_{ri}=\sum_{j=1}^{N}a_{ij}({\bar{r}}_{i}-{\bar{r}}_{j})+b_{i}{\bar{r}}_{i},i=1\ldots N$$

The local position error reflects the positional interaction between agent iii and its neighbors through the adjacency weight $a_{ij}$, as well as the agent’s own tracking error ${\bar{p}}_{i}$ (with self-weight $b_{i}$). Similarly, the local velocity error characterizes the coupling relationships in velocity.

We defined the following vectors:

(41) $$\bar{P}={\left[{\bar{p}}_{1}^{T},\ldots ,{\bar{p}}_{N}^{T}\right]}^{T},\bar{R}={\left[{\bar{r}}_{1}^{T},\ldots ,{\bar{r}}_{N}^{T}\right]}^{T}$$

(42) $$\varepsilon_{1}={\left[e_{p1}^{T},..,e_{pN}^{T}\right]}^{T},\varepsilon_{2}={\left[e_{r1}^{T},..,e_{rN}^{T}\right]}^{T}$$

(43) $$G_{p}={\left[g_{1}{\left(p_{1}\right)}^{T},..,g_{N}{\left(p_{N}\right)}^{T}\right]}^{T}$$

The global vectors of position, velocity, tracking errors, and local errors for all agents were then be compactly expressed as follows:

(44) $$\varepsilon_{1}=(L+B)⊗I_{n}\cdot \bar{P}$$

(45) $$\varepsilon_{2}=(L+B)⊗I_{n}\cdot \bar{R}$$

The velocity vector $\bar{R}$ can be rewritten in terms of the proportional valve function as follows:

(46) $$\begin{array}{l} \bar{R}=[{\bar{r}}_{1}^{T},\ldots ,{\bar{r}}_{N}^{T}{]}^{T}=\left[\begin{array}{c} \Phi (u_{1},p_{t\arg et,1})\\ \vdots \\ \Phi (u_{N},p_{t\arg et,\;N}) \end{array}\right]\\ =\Phi (U,p_{t\arg et}) \end{array}$$

By taking the time derivative, we obtained the followingd:

(47) $${\dot{\varepsilon }}_{1}=\varepsilon_{2}$$

(48) $${\dot{\varepsilon }}_{2}=(L+B)⊗I_{n}\cdot [G(p)+\dot{\Phi }(U,p_{t\arg et})]$$

According to Equations (3) and (12), we introduced two diagonal matrices to uniformly represent all follower cylinders:

Velocity control sensitivity matrix:

(49) $$K(P_{target})=diag(kP_{s}-P_{target,i})$$

Pressure coupling term matrix:

(50) $$H(U,P_{target})=diag(\frac{ku_{i}}{2\sqrt{P_{s}-P_{t\arg et,i}}})$$

Thus, the final expression becomes:

(51) $${\dot{\varepsilon }}_{2}=(L+B)⊗I_{n}\cdot \left[\begin{array}{l} G(p)+K(p_{t\arg et})\cdot \dot{U}\\ -H(U,P_{t\arg et})\cdot {\dot{P}}_{t\arg et} \end{array}\right]$$

To capture the nonlinear characteristics of proportional valve control, this section introduces a more realistic proportional valve control model $\Phi (u_{i},p_{t\arg et,i})$ to accurately characterize the system dynamics.

With the sliding surface established, the remaining task was to design a distributed control input that enforces finite-time reaching of this manifold while compensating for lumped uncertainties and external disturbances using only local and neighbor information. This motivates the controller structure developed in the next subsection.

### 4.6. Sliding Surface for Position Control

Design Rationale: The proposed distributed control law was constructed in a modular form to explicitly reflect the roles of different components. First, a nominal feedback term was introduced to stabilize the known part of the error dynamics and to shape the transient response on the sliding surface. Second, a robust term was incorporated to counteract the lumped uncertainty/disturbance that arises from friction, load variations, and unmodeled nonlinearities. Third, a finite-time reaching mechanism was embedded through a nonlinear reaching law so that the sliding variable is driven to the manifold rapidly. Finally, an adaptive adjustment mechanism was included to relax the need for exact a priori bounds on the uncertainties while maintaining boundedness of all closed-loop signals. This structure enables both practical implementation and the transparent Lyapunov-based proof of finite-time reaching in Section 4.8.

By integrating the tracking errors $\varepsilon_{1}$ and $\varepsilon_{2}$, the sliding surface was constructed. Specifically, $\varepsilon_{1}$ and $\varepsilon_{2}$ represent the deviation of the actual system state from the desired reference state from different perspectives and serve as key indicators of tracking performance. The diagonal matrices $C_{1},C_{2},C_{3}$$\left(c_{ii}>0\right)$ were introduced to assign adjustable weights to different agents or state components. A larger coefficient ($c_{ij}$) was used for critical state variables to enable faster convergence to the desired state and to enhance control precision.

Based on the established error dynamics, the sliding surface can be defined as follows [14]:

(52) $$S=C_{1}⊗I_{m}\cdot sign(\varepsilon_{1})+C_{2}⊗I_{m}\cdot \varepsilon_{2}+C_{3}⊗I_{m}\cdot \varepsilon_{1}$$

where $C_{1}=\mathrm{diag}(c_{11},\ldots ,c_{1n}),C_{2}=\mathrm{diag}(c_{21},\ldots ,c_{2n})$, $C_{3}=\mathrm{diag}$$\left(c_{31},\ldots ,c_{3n}\right)$. When β≫1, the hyperbolic tangent function can approximate the discontinuous sign function, i.e., $tanh(\beta \varepsilon_{1})\approx sign(\varepsilon_{1})$. Compared with the discontinuous sign function, tanh(⋅) is smooth and differentiable, which avoids chattering while retaining the rapid convergence property. This also facilitates mathematical analysis and reduces the risk of instability. Thus, the approximated sliding surface is as follows [1,13]:

(53) $$\bar{S}=C_{1}⊗I_{m}\cdot \tanh(\beta \varepsilon_{1})+C_{2}⊗I_{m}\cdot \varepsilon_{2}+C_{3}⊗I_{m}\cdot \varepsilon_{1}$$

Although the above control structure ensures fast reaching behavior in theory, practical hydraulic systems may suffer from chattering and unknown disturbance bounds. Therefore, we introduce an adaptive update mechanism and a continuous approximation of the switching action in the next subsection to enhance robustness and implementation smoothness.

### 4.7. Finite-Time Controller Design

To further enhance robustness without requiring exact bounds of uncertainties, this subsection introduces the adaptive update mechanism and the reaching law used in the proposed scheme. The adaptive terms are designed to adjust the control amplitude online, whereas the continuous approximation (e.g., tanh/saturation) mitigates chattering in practical implementations. Together, these components ensure reliable disturbance rejection while maintaining the desired finite-time convergence characteristics.

According to Equation (19), the time derivative of the sliding surface is given by the following:

(54) $$\begin{array}{l} \dot{\bar{S}}=C_{1}⊗I_{m}\cdot \beta [(I-tanh^{2}(\beta \varepsilon_{1}))\cdot \varepsilon_{2}]\\ +C_{2}⊗I_{m}\cdot {\dot{\varepsilon }}_{2}+C_{3}⊗I_{m}\cdot {\dot{\varepsilon }}_{1} \end{array}$$

To ensure that the system state reached and maintained the sliding manifold, two control terms were introduced: $K_{1}⊗I_{m}\cdot \mathrm{sign}(\bar{S})$ and $K_{2}⊗I_{m}\cdot \bar{S}$, which regulate the control amplitude. The control condition is defined as follows:

(55) $$\begin{array}{l} 0=C_{1}⊗I_{m}\cdot \beta [(I-tanh^{2}(\beta \varepsilon_{1}))\cdot {\dot{\varepsilon }}_{1}]\\ +C_{2}⊗I_{m}\cdot (L+B)⊗I_{n}\cdot (G+K\cdot \dot{U}-H\cdot {\dot{P}}_{t\arg et})\\ +C_{3}⊗I_{m}\cdot {\dot{\varepsilon }}_{1}+K_{1}⊗I_{m}\cdot sign(\bar{S})+K_{2}⊗I_{m}\cdot \bar{S} \end{array}$$

Therefore, the control law $\dot{U}$ is designed as follows:

(56) $$\dot{U}=-K^{-1}(p_{t\arg et})\cdot {\left(L+B\right)}^{-1}\cdot \left[\begin{array}{l} C_{1}\beta (I-tanh^{2}(\beta \varepsilon_{1}))\cdot {\dot{\varepsilon }}_{1}\\ +C_{3}\cdot {\dot{\varepsilon }}_{1}+C_{2}\cdot (G-H\cdot {\dot{P}}_{target})\\ +K_{1}\cdot sign(\bar{S})+K_{2}\cdot \bar{S} \end{array}\right]$$

The controller design integrates the advantages of adaptive terminal sliding mode control with deep reinforcement learning [13], enhancing adaptability to disturbances and nonlinearities in hydraulic systems. The dual control terms (discontinuous and linear) are typical in finite-time sliding mode control [10,11], ensuring both fast convergence and steady-state accuracy.

Having specified the complete controller and adaptive laws, we now provide a rigorous finite-time stability analysis to verify that the proposed distributed scheme guarantees boundedness of all signals and achieves the desired consensus tracking objective under the stated assumptions.

### 4.8. Control System Architecture

**Figure 2:**
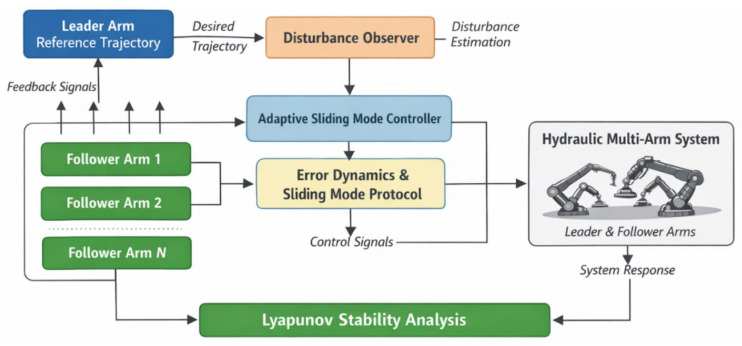
This figure illustrates the control structure for coordinating multiple hydraulic arms. The leader arm sets the desired trajectory, which is followed by the follower arms. The disturbance observer estimates external disturbances, and the adaptive sliding mode controller ensures synchronization and robustness by using error dynamics and the sliding mode protocol. The Lyapunov stability analysis guarantees finite-time convergence of the system.

In this section, we present the overall structure of the proposed control system. The following block diagram illustrates the flow of information and control signals across different components in the system. As shown in Figure 2, the control system consists of several key modules: the sliding mode controller, the disturbance observer, and the adaptive gain adjustment mechanism. Each module plays a vital role in ensuring fast synchronization and robustness in the hydraulic multi-arm system.

The sliding mode controller computes the tracking errors and applies the necessary control input, whereas the disturbance observer estimates the unknown disturbances and compensates for them in real time. The adaptive mechanism adjusts the control gains based on the estimated disturbances to ensure robustness against varying system parameters.

### 4.9. Finite-Time Stability Analysis

This subsection provides a rigorous finite-time stability analysis for the closed-loop distributed system. A Lyapunov function was constructed to show that all signals remain bounded and that the sliding variable reaches the manifold within a finite time under the proposed controller and adaptive laws. Finally, we translated the reaching result into the consensus tracking objective, thereby completing the logical link from controller design to synchronized position/velocity regulation stated in Section 4.1.

To analyze the finite-time convergence of the proposed sliding mode consensus controller, a Lyapunov candidate function was chosen as follows [10]:

(57) $$V=\frac{1}{2}{\bar{S}}^{T}\bar{S}$$

Taking the time derivative of V yields the following:

(58) $$\dot{V}=\frac{d}{dt}(\frac{1}{2}{\bar{S}}^{T}\bar{S})={\bar{S}}^{T}\dot{\bar{S}}$$

Based on the prior modeling and controller design, the dynamic expressions of the sliding surface $\dot{\bar{S}}$ and the control input $\dot{U}$ have already been derived in Equation (20) and Equation (22), respectively. By substituting these into $\dot{V}$, we obtain the following:

(59) $$\begin{array}{l} V={\bar{S}}^{T}\dot{\bar{S}}=-{\bar{S}}^{T}(C_{2}⊗I_{m})K_{1}sign(\bar{S})\\ -{\bar{S}}^{T}(C_{2}⊗I_{m})K_{2}\bar{S}+{\bar{S}}^{T}\Psi (t) \end{array}$$

where $K_{1}$, $K_{2}$ are diagonal positive definite gain matrices; Ψ(t) represents lumped system uncertainties and residual terms from $\varepsilon_{2}$, G(p), and ${\dot{P}}_{target}$, and is assumed to be bounded: ∥Ψ(t)∥≤δ, with known δ>0.

By applying norm inequalities, the Lyapunov function derivative is bounded as follows [12,29]:

(60) $$\dot{V}\leq -(\lambda_{1}-\delta )∥\bar{S}∥-\lambda_{2}∥\bar{S}{∥}^{2}$$

where $\lambda_{1}=min(c_{2,i}k_{1,i})$, $\lambda_{2}=min(c_{2,i}k_{2,i})$, $c_{2,i}$ denotes the weighting factor for the i-th subsystem in the sliding surface design, whereas $k_{1,i}$ and $k_{2,i}$ represent the corresponding gain elements in the discontinuous and linear stabilizing control terms, respectively. Both are drawn from the positive definite diagonal matrices $K_{1}$ and $K_{2}$. These definitions of $\lambda_{1}$ and $\lambda_{2}$ facilitate unified lower-bound estimation in the Lyapunov-based convergence analysis.

To connect this bound to the Lyapunov function V (and thus verify finite-time stability), we used the definition of V in Equation (57). Since $V=\frac{1}{2}∥\bar{S}{∥}^{2}$, we directly derived $∥\bar{S}∥=\sqrt{2V}$ and $∥\bar{S}{∥}^{2}=2V$. Substituting these relationships into Equation (60) transforms the bound into a constraint on V itself, as follows:

(61) $$\dot{V}\leq -(\lambda_{1}-\delta )\sqrt{2V}-\lambda_{2}\cdot 2V$$

We simplified this by defining $a=(\lambda_{1}-\delta )\sqrt{2}$ and $b=2\lambda_{2}$, leading to the comparison lemma form, as follows:

(62) $$\dot{V}\leq -a\sqrt{V}-bV,\;a=(\lambda_{1}-\delta )\sqrt{2},\;b=2\lambda_{2}$$

This inequality satisfies the core mathematical condition for finite-time stability: for a positive definite Lyapunov function, V, if $\dot{V}\leq -kV^{\delta }$ (where 0 < δ < 1), the system converges to zero in finite time. Here, $\sqrt{V}=V^{1/2}$ (so δ=1/2∈(0,1)) and k=min(a,b)>0, confirming that Equation (61) meets the finite-time stability criterion. To compute the finite convergence time, we integrated Equation (61) from the initial time t = 0 (with an initial value of V(0)) to the time T when V(T)=0 (system convergence), as follows:

(63) $$\int_{V(0)}^{0}\frac{dV}{a\sqrt{V}+bV}\leq \int_{0}^{T}dt$$

Let $u=\sqrt{V}$ (so dV=2udu). As such, the left integral simplifies to the following:

(64) $$2\int_{\sqrt{V(0)}}^{0}\frac{du}{a+bu}=\frac{2}{b}ln(\frac{a}{a+b\sqrt{V(0)}})$$

For practical hydraulic multi-arm systems, the initial error is small (i.e., $b\sqrt{V(0)}\ll a$, so $ln(\frac{a}{a+b\sqrt{V(0)}})\approx -\frac{b\sqrt{V(0)}}{a}$. Substituting this approximation back, we obtained the bounded convergence time, as follows:

(65) $$T\leq \frac{2\sqrt{V(0)}}{\lambda_{1}-\delta }$$

We conclude that the Lyapunov function vanishes in finite time, as follows:

(66) $$V(t)=0,\forall t\geq T\leq \frac{2\sqrt{V(0)}}{\lambda_{1}-\delta }$$

Thus, the system state ($\bar{S}(t)$) converges to the sliding manifold in finite time, achieving precise cooperative coordination among hydraulic arms.

## 5. Simulation Results: Position Tracking

This section validates the proposed position/height synchronization controller through a simulation study designed to reflect practical hydraulic deployment conditions. To ensure clarity and logical continuity, we first specify the simulated multi-arm platform, reference trajectory, and initial conditions, and then evaluate the closed-loop response under representative uncertainty conditions. The results are reported in a consistent manner by jointly examining (i) tracking/synchronization trajectories, (ii) tracking errors, and (iii) control inputs, so that convergence behavior, robustness, and actuation smoothness can be assessed in an integrated way.

A simulation was conducted on a coal bunker cleaning robot with one leader and three followers. Each actuator follows a second-order model, with m=40 kg, c=0.5 N⋅s/m, and k=1000 N/m, $F_{L}=0.6\;N$.

The leader broadcasts a sinusoidal reference input, as follows:

(67) $$u_{0}(t)=0.2\cdot \sin(\frac{2\pi }{12}t)$$

Initial positions: $p_{0}(0)=1$, $p_{1}(0)=2$, $p_{2}(0)=4$, and $p_{3}(0)=0$.

The sliding surface adopted is as follows:

(68) $$S_{i}=C_{1}\cdot tanh(\beta e_{p,i})+C_{2}\cdot e_{r,i}+C_{3}\cdot e_{p,i},i=1,2,3$$

Parameters: $C_{1}=1.2$, $C_{2}=1.2$, $C_{3}=2.5$, $K_{1}=6$, $K_{2}=10$, and β=2.0.

States $p_{1}$ and $r_{1}$ were updated via a discrete second-order loop.

**Figure 3:**
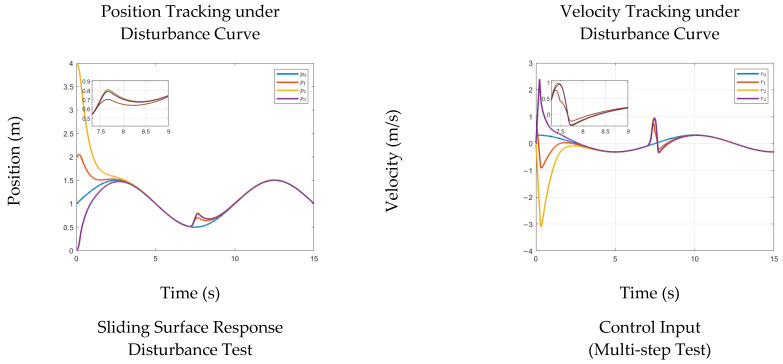

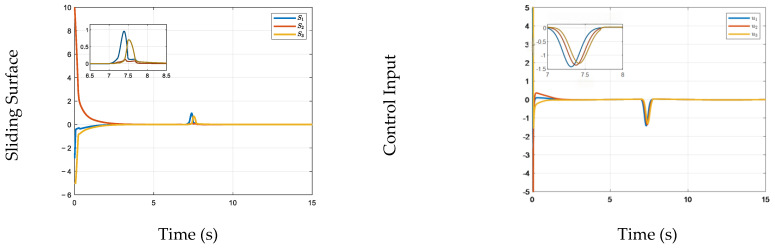
Multi-Joint Tracking and Control Performance under Disturbance Conditions. Joint Position, Velocity, Sliding Surface, and Control Input under Sinusoidal Reference and Disturbance Conditions.

We first report the nominal synchronization response and tracking accuracy, and then examine how the closed-loop system behaves when disturbances or parameter variations are introduced. The disturbance-test results of the sliding-surface response and control input are shown in Figure 3.

Overall, the simulations under both disturbance injection and parameter perturbation confirm that the proposed strategy achieves reliable synchronization with fast and well-damped transients, and that its performance remains consistent under the practical uncertainty sources typical of hydraulic actuation. Having established the effectiveness of the proposed controller in the above scenarios, the next section provides a direct comparison with baseline methods to further quantify the relative advantages in tracking accuracy and control effort.

## 6. Disturbance Observer-Based Control (DOBC)

### 6.1. Introduction to the DOBC Framework

Disturbance Observer-Based Control (DOBC) provides an alternative methodology for handling uncertainties in multi-arm systems by actively estimating and compensating for lumped disturbances. Although the proposed FTSMC strategy relies on sliding mode robustness, DOBC employs a model-based observer to reconstruct disturbances in real-time. This section presents a complete derivation of the DOBC scheme, stability analysis, and simulation validation to establish a rigorous benchmark for comparative evaluation in Section 7.

### 6.2. Dynamic Modeling with Disturbance

By extending the Euler–Lagrange dynamics from Equation (1) to explicitly include disturbance effects, the following is acheived:

(69) $$M_{i}(q_{i}){\ddot{q}}_{i}+C(q_{i},{\dot{q}}_{i}){\dot{q}}_{i}+G_{i}(q_{i})=\tau_{i}+d_{i}(t)$$

where $d_{i}(t)$ represents the lumped disturbance encompassing unmodeled dynamics, friction, and external perturbations. This is reformulated into a state-space form using Equations (3)–(5), as follows:


(70) $${\dot{x}}_{i}=v_{i},{\dot{v}}_{i}=M_{i}^{-1}(q_{i})[\tau_{i}-C(q_{i},{\dot{q}}_{i})v_{i}-G_{i}(q_{i})+d_{i}(t)]$$

**Assumption** **3.** *The disturbance* $d_{i}(t)$ *and its derivative are bounded, satisfying* $∥d_{i}(t)∥\;\leq d_{max}\;and\;∥{\dot{d}}_{i}(t)∥\;\leq {\bar{d}}_{max}$.

In the simulation study, the injected external disturbances are step-like and piecewise constant. Therefore, ${\dot{d}}_{i}(t)$ may exhibit impulsive behavior at the switching instants. In this work, the bounded-rate condition in Assumption 3 is interpreted in a practical sense, i.e., $d_{i}(t)$ is bounded and of bounded variation, with finitely many discontinuities, and ${\dot{d}}_{i}(t)$ is bounded almost everywhere except at those instants.

Rearranging (70), the lumped disturbance can be expressed as follows:

(71) $$d_{i}(t)=M_{i}(q_{i}){\dot{v}}_{i}+C_{i}(q_{i},{\dot{q}}_{i})v_{i}+G_{i}(q_{i})-\tau_{i}$$

### 6.3. Disturbance Observer Design

The ideal disturbance observer requires acceleration measurements, which is often impractical. To overcome this limitation, we developed an auxiliary variable approach.

Step 1—Ideal Observer Formulation:

A standard first-order disturbance observer was chosen, as follows:

(72) $${\dot{\hat{d}}}_{i}=L_{i}[d_{i}(t)-{\hat{d}}_{i}],L_{i}>0$$

The direct use of (71) requires ${\dot{v}}_{i}={\ddot{q}}_{i}$, which is often unavailable.

Step 2—Auxiliary Variable Design:

We introduced an auxiliary variable $z_{i}$ and function $p_{i}(v_{i})$ as follows:

(73) $${\hat{d}}_{i}(t)=z_{i}+p_{i}(v_{i})$$

We chose the following:

(74) $$p_{i}(v_{i})=L_{i}M_{i}(q_{i})v_{i}$$

so that the acceleration term cancels in the implementation.

We differentiated (73) and (74), as follows:

(75) $${\dot{\hat{d}}}_{i}={\dot{z}}_{i}+L_{i}{\dot{M}}_{i}(q_{i})v_{i}+L_{i}M_{i}(q_{i}){\dot{v}}_{i}$$

Step 3—Practical Observer Dynamics (Acceleration-Free):

We substituted (71) into (72), as follows:

(76) $${\dot{\hat{d}}}_{i}(t)=L\left(M_{i}(q_{i}){\dot{v}}_{i}+C_{i}(q_{i},{\dot{q}}_{i})v_{i}+G_{i}(q_{i})-\tau_{i}-{\hat{d}}_{i}\right)i$$

We equated (75) and (76), and used ${\hat{d}}_{i}=z_{i}+L_{i}M_{i}(q_{i})v_{i}$, as follows:

$${\dot{z}}_{i}+L_{i}{\dot{M}}_{i}(q_{i})v_{i}+L_{i}M_{i}(q_{i}){\dot{v}}_{i}=L_{i}(M_{i}(q_{i}){\dot{v}}_{i}+C_{i}(q_{i},{\dot{q}}_{i})v_{i}+G_{i}(q_{i})-\tau_{i}-z_{i}-L_{i}M_{i}(q_{i})v_{i})$$

We cancelled the $L_{i}M_{i}(q_{i}){\dot{v}}_{i}$ terms to obtain the correct acceleration-free implementation, as follows:

(77) $${\dot{z}}_{i}=-L_{i}z_{i}+L_{i}^{2}M_{i}(q_{i})v_{i}+L_{i}(C_{i}(q_{i},{\dot{q}}_{i})v_{i}+G_{i}(q_{i})-\tau_{i})-L_{i}{\dot{M}}_{i}(q_{i})v_{i})$$

The disturbance estimate is as follows:

(78) $${\hat{d}}_{i}(t)=z_{i}+L_{i}M_{i}(q_{i})v_{i}$$

### 6.4. Lyapunov Stability Analysis

We defined the estimation error as follows:

(79) $${\tilde{d}}_{i}=d_{i}-{\hat{d}}_{i}$$

From (72),

(80) $${\dot{\tilde{d}}}_{i}={\dot{d}}_{i}-{\dot{\hat{d}}}_{i}={\dot{d}}_{i}-L_{i}(d_{i}-{\hat{d}}_{i})={\dot{d}}_{i}-L_{i}{\tilde{d}}_{i}$$

**Theorem** **1 (ISS/Ultimate Boundedness).** *Under Assumption 3, the disturbance estimation error* ${\tilde{d}}_{i}(t)$ *is uniformly ultimately bounded. Moreover, its ultimate bound can be made arbitrarily small by increasing* $L_{i}$.

**Proof.**  Consider the Lyapunov function

(81) $$V_{d}=\frac{1}{2}{\tilde{d}}_{i}^{T}{\tilde{d}}_{i}$$

Differentiate using (80):

(82) $${\dot{V}}_{d}={\tilde{d}}_{i}^{T}({\dot{d}}_{i}-L_{i}{\tilde{d}}_{i})\leq \left\|{\tilde{d}}_{i}\right\|\left\|{\dot{d}}_{i}\right\|-L_{i}{\left\|{\tilde{d}}_{i}\right\|}^{2}\leq \left\|{\tilde{d}}_{i}\right\|{\dot{d}}_{\max}-L_{i}{\left\|{\tilde{d}}_{i}\right\|}^{2}$$

By using ${\dot{\tilde{d}}}_{i}(t)={\dot{d}}_{i}-{\dot{\hat{d}}}_{i}$ and substituting Equation (76), the following is obtained:

(83) $${\dot{V}}_{d}={\tilde{d}}_{i}^{T}M_{i}(q_{i}){\dot{d}}_{i}+{\tilde{d}}_{i}^{T}L_{i}M_{i}(q_{i}){\tilde{d}}_{i}+\frac{1}{2}{\tilde{d}}_{i}^{T}{\dot{M}}_{i}(q_{i}){\tilde{d}}_{i}$$

Hence, whenever $\left\|{\tilde{d}}_{i}\right\|>{\dot{d}}_{\max}/L_{i}$, one has ${\dot{V}}_{d}<0$, implying that ${\tilde{d}}_{i}(t)$ enters and remains in the set $\left\|{\tilde{d}}_{i}(t)\right\|\leq \frac{{\dot{d}}_{\max}}{L_{i}}$ for all sufficiently large t.

This proves uniform ultimate boundedness (ISS w.r.t. input ${\dot{d}}_{i}$). □

**Corollary** **1 (exponential convergence to zero).** *If* ${\dot{d}}_{i}(t)\equiv 0$ *(i.e.,* $d_{i}(t)$ *is constant), then (80) reduces to* ${\dot{\tilde{d}}}_{i}(t)=-L_{i}{\tilde{d}}_{i}$*, and* ${\tilde{d}}_{i}(t)$ *converges to zero exponentially with rate* $L_{i}$.

### 6.5. Simulation and Comparative Analysis

To rigorously validate the effectiveness, robustness, and practical value of the designed Disturbance Observer-Based Control (DOBC) scheme as a benchmark for comparison, comprehensive simulations were conducted under the same simulation platform and test scenarios as those used for the proposed FTSMC method in Section 5. The configuration included the identical three-arm hydraulic system model, the same sinusoidal reference trajectory ($u_{0}(t)=0.2sin(2\pi t/12)$), and the injection of identical step-like bounded external disturbances at the same time instants (3, 6, 9, 12, and 15 s).

**Figure 4:**
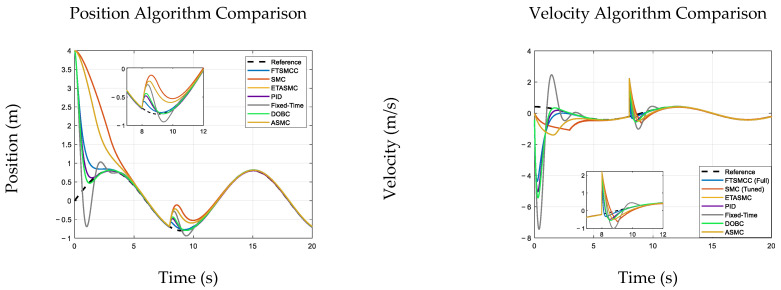
Comparison of Multiple Algorithms for Position and Velocity Tracking under Disturbance. This figure compares the tracking performance of seven control algorithms—FTSMCC, SMC, ETASMC, PID, FTCC, DOBC, and ASMC—against a reference trajectory under disturbances. The left subfigure shows position tracking, whereas the right displays the velocity response. The insets highlight recovery during disturbance, illustrating differences in convergence speed and disturbance rejection among the compared methods.

Quantitative results, including the root mean square error (RMSE) of tracking, recovery time (ST2), and control energy consumption, are summarized in Table 2, Table 3 and Table 4, and Figure 4 of Section 7 (Algorithm Comparison and Analysis). The simulation results show that DOBC achieves improved steady-state tracking accuracy compared with conventional Sliding Mode Control (SMC) and Adaptive Sliding Mode Control (ASMC) (RMSE = 0.07614) and maintains consistent performance under various parameter perturbations (see Table 4). These observations are consistent with the theoretical property of DOBC that the disturbance estimation error is uniformly ultimately bounded, and that the bound can be reduced by increasing the observer gain L_i_. Although the control energy efficiency of DOBC is slightly lower than that of FTSMC, the above verification confirms its effectiveness as an actively compensating benchmark controller and provides a solid basis for the fair and quantitative comparison in Section 7.

## 7. Comparative Analysis

To address reproducibility and clarify the literature basis of the comparative study, this section compares the proposed FTSMCC with six baselines that are explicitly implemented in the simulation code: SMC, ETASMC (event-triggered adaptive sliding mode), PID, Fixed-Time (fixed-time reaching sliding-mode baseline), DOBC, and ASMC. The baseline designs are grounded in representative references: classical sliding-mode control originates from Utkin’s variable-structure formulation [33]; PID follows standard PID analysis and tuning practice [35]; DOBC is based on disturbance–observer design principles and the stability considerations summarized in [38]; and adaptive/robust sliding-mode concepts are consistent with [37]. Fixed-time stability is theoretically supported by Polyakov’s fixed-time stabilization results [39] and related fixed-time consensus/control developments [40]. Event-triggered adaptive sliding-mode control is motivated by the event-triggered adaptive SMC literature (e.g., [41]).

All controllers were evaluated under identical simulation settings to ensure a fair comparison. SMC (Sliding Mode Control): switching gain = 1.2 and the sliding surface coefficient = 0.8; all baseline controllers were implemented exactly as in the released simulation code. For the evaluated follower, the tracking errors were $e_{p}=p-p_{0}$ and $e_{v}=v-v_{0}$, and the common sliding variable was $s=e_{v}+\lambda e_{p}$.

SMC (Utkin-type) [33]: $u=-\lambda e_{v}-\eta sgn(s)$.

ETASMC (event-triggered + adaptive, motivated by [41]): the control was updated only at triggering instants and was otherwise held constant (zero-order hold); at an update instant, $u=-\lambda ev-\hat{\eta }tanh(s/\phi )$, where $\hat{\eta }$ is adapted online and the triggering rule depends on the change of $\left[e_{p},e_{v}\right]$.

PID [35]: $u=-K_{p}e_{p}-K_{i}\int e_{p}dt-K_{d}e_{v}$.

Fixed-Time reaching SMC baseline [39,40]: $u=-\lambda e_{v}-k_{1}{\left|s\right|}^{\alpha }sgn(s)-k_{2}{\left|s\right|}^{\beta }sgn(s)$, with 0<α<1<β.

DOBC [38]: a disturbance observer estimates $\hat{d}$ (acceleration-free realization with an internal state) and the controller compensates it via $u=-k_{p}e_{p}-k_{v}e_{v}-\hat{d}$.

ASMC [37]: an adaptive/robust sliding-mode variant using boundary-layer switching ($u=-\lambda e_{v}-\hat{\eta }\;\tanh(s/\phi )$), with an online gain update.

All controllers were simulated with the same sampling period (Δt=0.01s), reference trajectory, and disturbance injection. Tracking accuracy was evaluated using RMSE computed from the original position error epe_pep. Settling time was reported in a dual-stage manner: ST1 (initial convergence) and ST2 (post-disturbance recovery) were defined as the first times when $\left|e_{p}\right|$ enters and remains within a tolerance band ($|e_{p}|\leq \varepsilon$) for at least a T_w_-second window (before and after the disturbance time t_d_, respectively). In this work, ε = 0.025 and T_w_ = 1 s were used for all methods.

RMSE was computed over the full simulation horizon as $RMSE=\sqrt{\frac{1}{T}\int_{0}^{T}{\left\|e(t)\right\|}^{2}dt}$. The settling time is reported in a dual-stage manner: ST1 denotes the first time instant when the tracking error enters and remains within a prescribed tolerance band around the reference (initial convergence), whereas ST2 measures the recovery time required for the error to re-enter and remain within the same band after the disturbance injection (post-disturbance recovery).

To evaluate multiple controllers under realistic conditions, the simulations considered two factors: persistent external disturbances and parameter uncertainties. A continuous sinusoidal disturbance was introduced to emulate environmental interference (e.g., vibration/load fluctuation), whereas key parameters (mass and damping) were perturbed (e.g., +30% mass and −30% damping) to represent variability/model mismatch. All controllers used identical reference trajectories to ensure a fair comparison. The control energy was defined as the L2 norm of the control input (hydraulic valve signal) to quantify actuation efficiency, calculated by $E=\int_{0}^{T}∥\tau (t){∥}^{2}dt$.

To better reflect implementation conditions, we further considered non-ideal factors, including actuator saturation and rate limits, measurement noise, and an N_d_-step sensing/communication delay. All controllers were evaluated under the same non-ideal settings for fairness.

**Figure 5:**
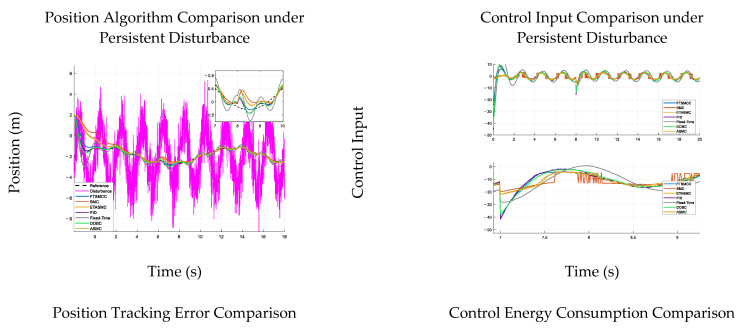

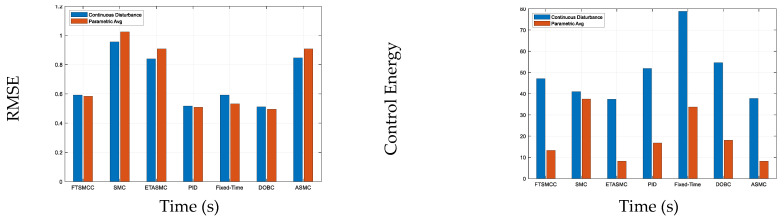
Performance Comparison of Control Algorithms under Persistent Disturbance and Parameter Variations. This figure presents a comprehensive comparison of seven control algorithms—FTSMCC, SMC, ETASMC, PID, FTCC, DOBC, and ASMC—under persistent external disturbances and parameter perturbations. The subfigures report tracking performance and control efforts under identical test conditions, enabling a fair assessment of robustness and energy consumption across different control strategies. Baseline controller structures follow [33,35,37,38,39,40,41].

Where T=15 s (simulation duration), τ(t) denotes the control input torque of the hydraulic arms, and the unit of E is $N^{2}\cdot s$. This definition is consistent with the energy evaluation standard for hydraulic control systems [5]. Figure 5 shows improved robustness and efficiency under disturbances and perturbations.

The results consistently demonstrate that FTSMCC provides the most practically favorable trade-off between robustness, disturbance recovery, and control efficiency across all test scenarios. In the dual-stage settling-time comparison (Table 3), although PID and DOBC achieve slightly smaller RMSE values in some cases (e.g., PID: 0.07803; DOBC: 0.07614 versus FTSMCC: 0.08277), FTSMCC achieves the fastest post-disturbance recovery, with ST2 = 1.10 s, which is substantially shorter than PID (4.71 s), SMC (3.54 s), ETASMC/ASMC (2.70 s), and also faster than Fixed-Time (2.04 s) and DOBC (2.10 s). This indicates that FTSMCC restores synchronization significantly more rapidly once external perturbations occur, which is critical for hydraulic multi-arm coordination under abrupt load changes and unmodeled disturbances.

Moreover, under the continuous disturbance tests (Table 3), FTSMCC achieves competitive tracking accuracy (RMSE = 0.5880) while maintaining lower control energy (47.08) than PID (53.39) and DOBC (54.47), and is dramatically lower than the Fixed-Time baseline (77.39). Under parameter perturbations (Table 4 and Table 5), FTSMCC exhibits high consistency: its RMSE remains nearly unchanged across all parameter cases (approximately 0.58448 → 0.58433) and its control energy is also highly stable and low (approximately 13.317 → 13.332). In contrast, SMC requires much higher energy (~37.5) while delivering the worst RMSE (~1.02), and Fixed-Time also incurs significantly higher energy (~33.7). Although ETASMC/ASMC consume less energy (~8.26), their tracking accuracy deteriorates markedly (RMSE ≈ 0.907), indicating a less favorable robustness–accuracy balance.

Overall, these comparisons confirm that FTSMCC is distinguished primarily by its superior disturbance recovery speed (minimum ST2) and its consistently stable performance under parametric uncertainties while keeping control efforts at a moderate and practical level. Therefore, FTSMCC is particularly suitable for coordinated hydraulic multi-arm deployment, where rapid resynchronization after disturbances and robust operation under uncertain dynamics are decisive requirements.

## 8. Discussion

In conclusion, the expanded benchmarking against PID, SMC, ETASMC, Fixed-Time, DOBC, and ASMC verifies that the proposed FTSMCC offers the most balanced performance for coordinated hydraulic multi-arm control. Although some baselines can match or slightly improve steady-state RMSE under specific settings, FTSMCC consistently delivers the fastest disturbance recovery (minimum ST2), enabling rapid re-synchronization after perturbations. In addition, FTSMCC maintains high robustness under continuous disturbances and parameter variations, with stable tracking behavior and moderate control effort, avoiding the high-energy demand observed in Fixed-Time/PID/DOBC and the accuracy degradation seen in ETASMC/ASMC. Overall, these results confirm that FTSMCC is a practically attractive solution when disturbance resilience, fast recovery, and efficiency are simultaneously required in hydraulic multi-arm deployment.

Experimental validation on a real multi-arm platform will be conducted as future work to further demonstrate practical feasibility under hardware constraints and safety requirements. Due to limited platform access within the current revision period, this revision focuses on strengthened simulation with implementation non-idealities (saturation/rate limits/noise/delay).

## Figures and Tables

**Table 1:** Nomenclature.

Symbol	Physical Meaning/Mathematical Definition	Dimension
$M_{i}(q_{i})$	Symmetric positive definite inertia matrix of hydraulic arms	N × N
$C_{i}(q,\dot{q})$	Coriolis and centrifugal force matrix	N × N
$G_{i}(q_{i})$	Gravitational term vector	N × 1
$\tau_{i}$	Control input torque	N × 1
$d_{i}(t)$	External disturbance of the i-th hydraulic arm	1 × 1
$c_{i}$	Positive gain of sliding surface for the i-th arm	1 × 1
$\bar{S}$	Distributed sliding mode variable	N × 1
$\lambda_{i}$	Finite-time gain for position control	1 × 1
γ	Adaptive update rate	1 × 1

Note: The subscript *j* denotes the *j*-th hydraulic arm/subsystem; *N* denotes the total number of hydraulic arms in the system; the dimension of each symbol is determined by the system scale *N*.

**Table 2:** Controller Performance Comparison (Dual-Stage Settling Time).

Controller	RMSE	ST1	ST2
FTSMCC	0.08277	3.25 s	1.10 s
SMC	0.16040	5.81 s	3.54 s
ETASMC	0.14122	7.00 s	2.70 s
PID	0.07803	1.50 s	4.71 s
Fixed-Time	0.07678	4.90 s	2.04 s
DOBC	0.07614	1.87 s	2.10 s
ASMC	0.14122	7.00 s	2.70 s

**Table 3:** Performance Table under Continuous Disturbance Tests.

Controller Name	RMSE (Position Error)	Control Energy
FTSMCC	0.5880	47.08
SMC	0.9396	41.21
ETASMC	0.8739	38.31
PID	0.5203	53.39
Fixed-Time	0.5881	77.39
DOBC	0.5112	54.47
ASMC	0.8491	37.88

**Table 4:** RMSE Table under Parameter Perturbation Tests.

Controller Name	Nominal	Nominal Mass +30%	Damping −30%	Both Parameters Varied
FTSMCC	0.58448	0.58444	0.58438	0.58433
SMC	1.0236	1.0239	1.0238	1.0234
ETASMC	0.90745	0.90727	0.90722	0.90709
PID	0.50683	0.50643	0.50630	0.50630
Fixed-Time	0.53205	0.53225	0.53230	0.53244
DOBC	0.49587	0.49583	0.49581	0.49578
ASMC	0.90745	0.90727	0.90722	0.90709

**Table 5:** Control Energy Table under Parameter Perturbation Tests.

Controller Name	Nominal	Nominal Mass +30%	Damping −30%	Both Parameters Varied
FTSMCC	13.317	13.324	13.326	13.332
SMC	37.514	37.529	37.529	37.534
ETASMC	8.2553	8.2618	8.2638	8.2685
PID	16.569	16.611	16.642	16.674
Fixed-Time	33.654	33.706	33.721	33.758
DOBC	18.055	18.066	18.069	18.077
ASMC	8.255	8.262	8.264	8.269

## Data Availability

The original contributions presented in this study are included in the article. Further inquiries can be directed to the corresponding author.
